# An Adenovirus-Based Recombinant Herpes Simplex Virus 2 (HSV-2) Therapeutic Vaccine Is Highly Protective against Acute and Recurrent HSV-2 Disease in a Guinea Pig Model

**DOI:** 10.3390/v15010219

**Published:** 2023-01-13

**Authors:** Mingming Wan, Xiao Yang, Jie Sun, Xue Ding, Zhijun Chen, Weiheng Su, Linjun Cai, Ali Hou, Bo Sun, Feng Gao, Chunlai Jiang, Yan Zhou

**Affiliations:** 1 National Engineering Laboratory for AIDS Vaccine, School of Life Sciences, Jilin University, Changchun 130012, China; 2 Key Laboratory for Molecular Enzymology and Engineering, The Ministry of Education, School of Life Sciences, Jilin University, Changchun 130012, China

**Keywords:** HSV-2, recombinant adenovirus, therapeutic vaccine

## Abstract

Genital herpes (GH) has become one of the most common sexually transmitted diseases worldwide, and it is spreading rapidly in developing countries. Approximately 90% of GH cases are caused by HSV-2. Therapeutic HSV-2 vaccines are intended for people already infected with HSV-2 with the goal of reducing clinical recurrences and recurrent virus shedding. In our previous work, we evaluated recombinant adenovirus-based vaccines, including rAd-gD2ΔUL25, rAd-ΔUL25, and rAd-gD2, for their potency as prophylactic vaccines. In this study, we evaluated these three vaccines as therapeutic vaccines against acute and recurrent diseases in intravaginal challenged guinea pigs. Compared with the control groups, the recombinant vaccine rAd-gD2ΔUL25 induced a higher titer of the binding antibody, and rAd-gD2 + rAd-ΔUL25 induced a higher titer of the neutralizing antibody. Both rAd-gD2ΔUL25 and rAd-gD2 + rAd-ΔUL25 vaccines significantly enhanced the survival rate by 50% compared to rAd-gD2 and reduced viral replication in the genital tract and recurrent genital skin disease. Our findings provide a new perspective for HSV-2 therapeutic vaccine research and provide a new technique to curtail the increasing spread of HSV-2.

## 1. Introduction

Herpes simplex virus (HSV), the earliest discovered human herpes virus, is the most important etiologic pathogen of genital herpes (GH). GH is the most common sexually transmitted disease worldwide, causing severe discomfort and pain in patients, accompanied by concerns about public hygiene [1]. In 2020, an estimated half a billion people worldwide have had genital herpes [2]. Moreover, HSV-2 can increase the risk of human immunodeficiency virus (HIV) infection by 1–3 fold [3]. HSV-2 infects hosts in many ways, with mucosal infections being the most common. Once HSV-2 infects host cells, the virus is transferred from the sensory nerve to the dorsal root ganglion in the central nervous system, resulting in latent infection throughout the lifetime of the host.

Currently, although many attempts have been developed, none of these vaccines have been approved for the prevention or treatment of HSV-2 [4,5,6]. Antiviral medicines, such as acyclovir [7], valacyclovir [8], penciclovir, famciclovir, and ganciclovir, are only used to reduce recovery time and to reduce viral shedding. However, antiviral drugs cannot eliminate the virus’s latent infection; therefore, there is an urgent need to develop an HSV-2 therapeutic vaccine.

Glycoprotein D (gD), one of the glycoproteins on the envelope of HSV-2 encoded by *US6* of HSV-2, can specifically bind to the host membrane receptor herpes virus entry mediator (HVEM) and Nectin-1; it is important in the process of host cell recognition by the virus [9]. The capsid of HSV-2 is a spherical icosahedron (T = 16) [10] encoded by *UL25*. UL25 is approximately 62 kDa and is highly conserved across the herpes family [11]. Many epitopes on UL25 have been proven to activate the CD8+ T cell response and trigger the production of IFN-γ, making it an ideal antigen candidate for a herpes vaccine [12]. It is increasingly believed that successful vaccines generate a robust T-cell response in addition to humoral immunity against viral glycoproteins [1,7]. One of the most efficient approaches to enhancing T-cell immunity is to develop a virus-based vaccine. Because they can induce potent transgene product-specific antibodies and T-cell responses, recombinant replication-defective adenovirus serotype 5 (Ad5) vectors are considered promising vaccine delivery modalities [13].

In our previous study, we evaluated the prophylactic efficacy of recombinant replication-defective adenoviruses in a mouse model, which was capable of expressing gD2 (1-306aa) (rAd-gD2), truncated ΔUL25 (310-585aa) (rAd-ΔUL25), and fusion protein gD2-ΔUL25 (rAd-gD2ΔUL25) [14]. Recombinant adenovirus-based vaccines showed higher reductions in mortality and stronger antigen-specific T-cell responses than formaldehyde-inactivated HSV-2 (FI-HSV-2) did. Moreover, the severity of genital lesions in mice immunized with rAd-gD2ΔUL25 was significantly decreased by eliciting IFN-secreting T-cell responses compared with that in the rAd-gD2 groups. Although rAd-gD2ΔUL25 and rAd-ΔUL25 both induced UL25-specific cellular immunity, rAd-ΔUL25 did not provide any protection against the lethal dose intravaginal HSV-2 challenge [14]. These results suggest that our recombinant adenoviruses might have potential as therapeutic vaccine candidates.

The murine model is beneficial for immunology assays because of the availability of reagents to measure T-cell responses. However, infections do not recur spontaneously in mice, rendering this model useless for studying the efficacy of therapeutic vaccines. Therefore, we chose a guinea pig genital infection model to evaluate the efficacy of these therapeutic vaccines. Based on the survival rate and cellular immune response produced by our recombinant adenoviruses, to verify whether the recombinant adenoviruses might have the potential as a therapeutic vaccine candidate, in this study, we evaluated the therapeutic efficacy of three types of adenovirus-based vaccines in guinea pig models against acute and recurrent diseases by measuring guinea pig humoral and cellular responses, pathological grade, and genital tract viral loading.

## 2. Materials and Methods

### 2.1. FI-HSV-2 Preparation

An HSV-2 strain G (VR-734, ATCC, Manassas, VA, USA) was propagated by infecting confluent Vero cells (African green monkey kidney cells, CCL-81, ATCC, Manassas, VA, USA) monolayers at a multiplicity of infection (MOI) of 0.1, incubated at 34 °C for 72 h, and subsequently harvesting the cell-associated virus, as described previously [15]. The pelleted virions were then treated with 0.1% formalin for 3 days at 37 °C. Successful inactivation of HSV-2 was confirmed by its inability to produce cytopathic effects (CPE) when incubated with Vero cells. Protein content was measured using a Bradford protein assay kit (Bio-Rad Laboratories, Hercules, CA, USA).

### 2.2. Amplification of Recombinant Adenovirus Vaccine

Three types of recombinant replication-defective adenoviruses (stored in our laboratory), which were capable of expressing gD2 (rAd-gD2), truncated ΔUL25 (310-585aa) (rAd-ΔUL25), and fusion protein gD2-ΔUL25 (rAd-gD2ΔUL25), were constructed and identified in our previous study [14] and were amplified in this study. To amplify the recombinant adenovirus, recombinant adenoviruses rAd-gD2, rAd-ΔUL25, and rAd-gD2ΔUL25 were used to infect 90% confluent HEK293 cells (human embryonic kidney 293, cat# CRL-1573, ATCC) at an MOI of 0.1. After confirming 90% of cells with CPE, the infected cells were collected with centrifugation and repeatedly exposed to liquid nitrogen and a 37 °C water bath for freeze-thawing. The freeze-thawed supernatant was mixed with 20% PEG8000/2.5 M NaCl at a ratio of 2:1, placed on ice for 1 h, and centrifuged at 12,500× *g* for 30 min at 4 °C before collecting the precipitate.

The virus-containing precipitate obtained in the previous step was slowly re-suspended in 5 mL CsCl (Dingguo Changsheng Biotechnology, Beijing, China) solution of 1.1 g/mL density. For preparing a discontinuous CsCl density gradient, 2 mL of 1.4 g/mL CsCl, 3 mL of 1.3 g/mL CsCl, and 5 mL of re-suspension supernatant were mixed and centrifuged at 6000× *g* for 3 h at 4 °C; the virus strip was aspirated and transferred to a dialysis card for dialysis at 4 °C overnight. The dialysate was changed every 6 h, and the samples were stored at −80 °C.

### 2.3. Expression of Recombinant Virus

Western blotting was used to verify the presence of gD2 protein, ΔUL25 protein, and gD2ΔUL25 protein [14]. HEK293 cells were harvested when most cells showed CPE, and cell lysates were obtained by freeze-thawing the cells twice.

Proteins obtained from the cell extracts were confirmed by western blotting. Expressed antigens (gD2, UL25, and gD2ΔUL25) were separated via electrophoresis on 13.5% SDS-PAGE under denaturing conditions and transferred to a nitrocellulose membrane (Bio-Rad, Hercules, CA, USA). The gD2 rabbit polyclonal antibody (produced in our laboratory) [14] and 1:1000 anti-HA tag antibody (Abcam, Inc., Shanghai, China) were used for the western blot assays as previously described [14]. The viral stocks were dialyzed and stored as aliquots at −80 °C until further use. The infectious titer (pfu/mL) was determined by TCID50 assay using HEK293 cells.

### 2.4. Guinea Pig Viral Challenge and Immunization

Seventy-two female guinea pigs (250–300 g) were obtained from the Changchun Institute of Biological Products (Changchun, China) and challenged intravaginally with an LD50 of 1 × 10^6^ PFU of HSV-2 virus per guinea pig [16]. After half of the guinea pigs died, the remaining guinea pigs were randomly divided into 6 groups (Table 1). Guinea pigs were immunized twice at 14 and 28 days post-infection (dpi). The recombinant adenoviruses rAd-gD2ΔUL25, rAd-gD2 + rAd-ΔUL25, rAd-gD2, and FI-HSV were administered intramuscularly in the respective groups, and acyclovir was administered orally every day for ten days from 14 dpi to 23 dpi. Recombinant adenoviruses rAd-gD2ΔUL25 and rAd-gD2 were used to immunize the animals at 1 × 10^8^ PFU per guinea pig, and recombinant adenovirus rAd-gD2, balanced mixed with rAd-ΔUL25, was used to immunize the animals at a total of 2 × 10^8^ PFU per guinea pig. FI-HSV2 with Freund’s complete adjuvant was used subcutaneously (s.c.) for the first immunization and with the incomplete form for the second immunization, at 12.5 μg per guinea pig [14]. The guinea pigs were monitored, and pathological development was scored daily for several days post-challenge.

Disease scoring was as follows: 0 = no visible lesion; 1 = redness or swelling; 2 = skin lesions or hair loss; 3 = urine and fecal retention and hind limb paralysis; 4 = death [17]. A nylon-tipped applicator was used to collect vaginal secretions every alternate day from 14 dpi to 56 dpi, and the swabs were stored at −80 °C until analysis. All immunization and challenge experiments were repeated thrice.

All animal care procedures and experimental methods conformed to the guidelines of the Institutional Animal Care and Use Committee.

### 2.5. Direct ELISA for gD-Specific IgG

Serum samples were collected from whole blood using centrifugation (3000 rpm, 30 min, 4 °C) on 0, 14, 28, and 42 dpi and stored as aliquots at −80 °C until use. After completing the immunization protocol, guinea pigs were bled, and specific antibodies against gD were tested by direct ELISA.

Recombinant gD protein, expressed in the *E. coli* system (stored in our laboratory), was used to coat a 96-well plate overnight at 4 °C at 0.5 μg/well. Bovine serum albumin (BSA) was used to block non-specific binding, and serum samples were incubated in the plate at 37 °C for 2 h. HRP-conjugated goat anti-guinea pig IgG (Abcam, Shanghai, China) was used as the secondary antibody at 1:10,000 dilution, incubated in the plate for 1 h at 37 °C, followed by the development of the plates using a tetramethyl benzidine substrate solution (TMB; Sigma, St. Louis, MO, USA), and absorbance was read at 450 nm. Antibody titer was defined as the reciprocal of the highest dilution that gave an absorbance at least twice that produced by the negative control.

### 2.6. Neutralizing Antibody Assay

Neutralizing activity of each immune serum sample was tested by a micro-neutralization assay. Briefly, serum samples were heated at 56 °C for 30 min. Thereafter, 200 PFU of the HSV-2 strain G stock was incubated with two-fold serially diluted serum, starting at 1:20 dilution at 37 °C for 1 h. After incubation, each sample was mixed in duplicate on a Vero cell and further incubated for 7 days. The neutralizing titer was defined as the highest serum dilution for which 50% of Vero cells exhibited cytopathic effects [14].

### 2.7. Cytokine Assay

Cytokines in serum were determined by ELISA (Mlbio, Shanghai, China). The cytokine assay was performed according to the manufacturer’s instructions. Briefly, serum samples were diluted 1:5 with dilution buffer and added to a pre-coated ELISA plate. Standards at different concentrations were also added to the pre-coated ELISA plates. HRP-conjugated antibodies were immediately added to the plate. After incubation at 37 °C for 60 min, color changes in the plates were detected using a 3,3′,5,5′-tetramethylbenzidine substrate.

### 2.8. Virus Shedding

For each group of guinea pigs, a sterile cotton swab was inserted into the vagina every alternate day, from 14 dpi to 56 dpi. For collecting vaginal secretions, the swab was placed in a sterile EP tube and soaked at 4 °C for 5 h with 200 μL HBSS solution (Gibco, Shanghai, China). A viral genome extraction kit (Qiagen, Hilden, Germany) was used for extracting HSV-2 genomic DNA from swabs, and real-time quantitative fluorescent PCR was used for detecting DNA copies.

HSV-2 loads in vaginal swabs were determined by qPCR targeting the HSV-2 gG2 gene; specific primers used for gG2 DNA were as follows: forward, 5′–CCCACACCCCAACACATC-3′ and reverse, 5′–CCAAGGCGACCAGACAAAC-3′ [18]. A ten-fold dilution of gG2-Teasy recombinant plasmid (stored in our laboratory) was used as the PCR standard template to quantify the HSV-2 copy number in samples. The target DNA was detected by qPCR using SYBR green dye and the conditions of 30-s denaturation at 94 °C followed by 40 two-step amplification cycles (5 s at 94 °C and 30 s at 63 °C). A standard curve for the virus was generated as previously described, containing 10^5^–10^0^ HSV-2 gG2 copies [19]. The PCR products were analyzed using the Bio-Rad CFX Manager^TM^, version 2.1. The limit of detection for HSV-2 was determined to be between 10^0^ and 10^1^ copies; vaginal swab samples were considered negative if they contained <5 copies of HSV-2 DNA per assay.

### 2.9. Statistical Analysis

Statistical analyses were performed using one-way analysis of variance (ANOVA) to compare the differences in t values between the experimental and control groups and Student’s *t*-test in GraphPad Prism 9.0 (GraphPad, San Diego, CA, USA). *p* < 0.05 was considered significant. All experiments were repeated in triplicate. Values were expressed as the mean ± standard deviation (SD). Statistical significance was considered to be *p* < 0.05, and the represented by asterisks was marked correspondingly in the figures, where * *p* < 0.05; ** *p*< 0.01, *** *p* < 0.001; **** *p* < 0.0001, ns: non-significant.

## 3. Results

### 3.1. Expression of Recombinant Viruses and Proteins

**Figure 1:**
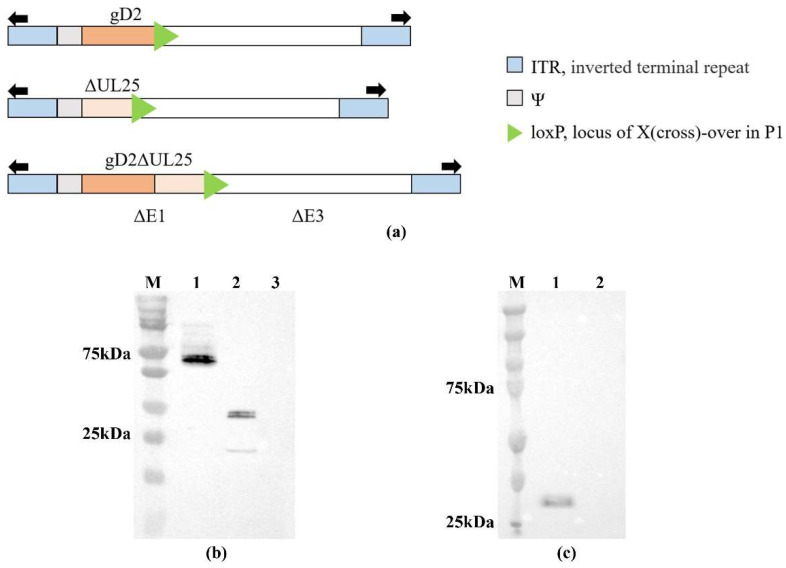
HEK293 cells were infected with recombinant adenoviruses or an empty adenovirus vector. Proteins produced from adenoviruses were assessed via Western blotting. (**a**) Schematic representation of recombinant adenoviruses. gD2, 1-306aa, ΔUL25, 310-585aa. (**b**) Western blot analysis of rAd-gD2ΔUL25 (Lane 1), rAd-gD2 (Lane 2), and empty adenovirus vector (Lane 3) using gD2 rabbit polyclonal antibody. gD2ΔUL25 protein is approximately 67 kDa, and gD2 protein is approximately 36 kDa. (**c**) Western blot analysis of rAd-ΔUL25 (Lane 1) and empty adenovirus vector (Lane 2) using anti-HA tag antibody. ΔUL25 protein is approximately 32 kDa. M, Precision Plus Protein Dual Color Standards.

A schematic representation of recombinant adenoviruses is shown in Figure 1a. Western blot analysis was performed to characterize gD2ΔUL25, gD2, and ΔUL25 proteins expression in HEK293 cell extracts (Figure 1b,c). A band of approximately 67 kDa was observed, which represented the gD2ΔUL25 protein (Figure 1b, lane 1), a band of approximately 36 kDa represented the gD2 protein (Figure 1b, lane 2), and another band of approximately 32 kDa was detected, representing the ΔUL25 protein (Figure 1c, lane 1). The molecular masses of gD2 and gD2ΔUL25 were 36 kDa and 67 kDa, respectively, which are slightly higher than the theoretical values of 32 kDa and 64 kDa because of the post-translational modifications of the gD2 protein. These results indicated that the rAd-gD2ΔUL25, rAd-gD2, and rAd-ΔUL25 recombinant viruses were successfully propagated in HEK293 cells.

### 3.2. Survival Rate of Guinea Pigs against LD50 HSV-2 Challenge

**Figure 2:**
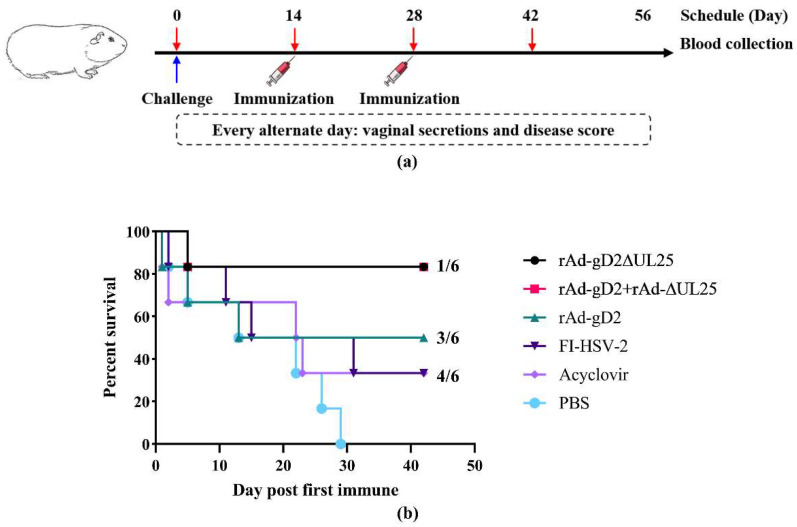
Experimental scheme and percent survival of guinea pigs in 42 days (**a**) Experimental scheme. Guinea pigs were challenged at day 0 and immunized with recombinant adenovirus vaccines, FI-HSV-2, or PBS twice at 1- and 28 dpi. Sera samples were collected at 0, 14, 28, and 42 dpi, and vaginal secretions were collected every alternate day from 0 to 56 dpi. (**b**) The *X*-axis shows days post-immunization, and *Y*-axis shows the percentage of surviving guinea pigs (*n* = 6 per group). The number to the right is the number of dead animals/total number of animals in each group. Both rAd-gD2ΔUL25 and rAd-gD2 + rAd-ΔUL25 had a survival percentage of 83.3%, rAd-gD2 had 50%, FI-HSV2 and acyclovir had 33.33%, and PBS had 0%.

Guinea pigs in all groups were challenged intravaginally with an LD50 of 1 × 10^6^ PFU of HSV-2 strain G. After half of the guinea pigs died, the remaining guinea pigs were randomly divided into 6 groups, and their disease symptoms were monitored once every two days, for 42 days after the first immunization. Immunization groups are shown in Table 1. The experimental scheme is shown in Figure 2a, and serum samples were collected on 0, 14, 28, and 42 dpi. As shown in Figure 2b, mock-immunized control animals that received PBS showed progressive disease symptoms, starting on day two post the first immunization, and nearly all of the animals died due to neurological diseases over the 29-day period. Animals immunized with FI-HSV-2 and the acyclovir-control group generated a protection rate of 33.3% against intravaginal challenge. In comparison to the positive control groups FI-HSV2 and acyclovir, groups immunized with rAd-gD2 showed 50% survival post-challenge, whereas those immunized with rAd-gD2ΔUL25 and rAd-gD2 + rAd-ΔUL25 showed the highest survival rate of 83.3%. These results indicated that gD2 with ΔUL25, both in fusion or in combination as the immunogen, could protect guinea pigs against the HSV-2 challenge and increase the survival rate compared to rAd-gD2.

### 3.3. Disease Scores

**Figure 3:**
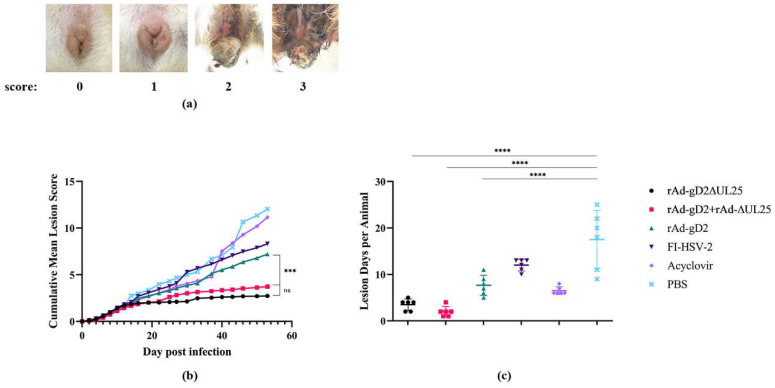
Recurrent disease in guinea pigs. (**a**) Images of different disease scores. 0 = no visible lesion; 1 = redness or swelling; 2 = skin lesions or hair loss; 3 = urine and fecal retention and hind limb paralysis; 4 = death. (**b**) Cumulative mean lesion scores in guinea pigs. All guinea pigs in the vaccinated groups showed reduced scores compared to the mock-immunized group and the group treated with acyclovir. rAd-gD2ΔUL25 and rAd-gD2 + rAd-ΔUL25 both significantly reduce disease lesions than rAd-gD2 group. (**c**) Days of animals with positive lesions are shown. rAd-gD2ΔUL25 and rAd-gD2 + rAd-ΔUL25 both significantly reduce positive lesions than rAd-gD2 group and acyclovir groups. The mean recurrence in rAd-gD2 + rAd-ΔUL25 group was 2 days and 3.5 days in the rAd-gD2ΔUL25 group. **** *p* < 0.0001; *** *p* < 0.001; ns, not statistically significant.

HSV-2 infection in guinea pigs induces recurrent lesions and viral shedding [18]. To examine the potential therapeutic effect of the vaccines, disease scores were determined every alternate day from the challenge to 56 dpi (Figure 3 and Table 2). Images of different disease scores are shown in Figure 3a. 0 = no visible lesion; 1 = redness or swelling; 2 = skin lesions or hair loss; 3 = urine and fecal retention and hind limb paralysis; 4 = death. The mock-immunized control animals experienced significantly more severe symptoms than the mock-immunized animals. All vaccinated groups showed lower scores than the mock-immunized group and the acyclovir-treated group. rAd-gD2ΔUL25 and rAd-gD2 + rAd-ΔUL25 both achieved cumulative mean scores of nearly 3, and a slight improvement (not significant) was observed in the cumulative mean disease score in rAd-gD2ΔUL25 with rAd-gD2 + rAd-ΔUL25 (Figure 3b). As shown in Table 2, each vaccination with recombinant adenovirus significantly reduced the disease score and inhibited the recurrence of the disease, and the addition of ΔUL25 in both the fusion and combined forms was particularly effective. The number of days for animals with positive lesions is shown in Figure 3c; the mean recurrence in the PBS group was 17.5 days, in the acyclovir group was 6.5 days, in the FI-HSV-2 group was 12 days, in the rAd-gD group was 7.7 days, in rAd-gD2 + rAd-ΔUL25 group was two days, and in the rAd-gD2ΔUL25 group was 3.5 days. No significant difference was observed between the rAd-gD2 + rAd-ΔUL25 group and the rAd-gD2ΔUL25 group. These results indicated that our recombinant adenovirus vaccines could significantly reduce the recurrence disease score, and the addition of UL25 could significantly reduce the lesion score compared with gD2 alone.

### 3.4. Quantitation of Viral Shedding

To examine the potential therapeutic effect of vaccines, quantitation of viral shedding was performed in HSV-2-infected guinea pigs. Vaginal HSV-2 shedding was measured by real-time PCR, as described in the Section 2, and samples were considered positive if they contained >5 copies of HSV-2 DNA per assay.

Table 3 shows the effects of adenovirus vaccines on viral shedding in HSV-2-infected guinea pigs. In this study, all animals in the groups showed a positive copy of HSV-2 DNA, which was considered a recurrent viral shedding day. Compared with the PBS group, the acyclovir group showed a decrease at 14–27 dpi; however, recurrence occurred after drug withdrawal, and acyclovir reduced the pathological score but did not significantly reduce virus shedding. By contrast, the immunization of guinea pigs with recombinant adenovirus caused a significant decrease in viral shedding and disease score compared with the acyclovir and FI-HSV-2 groups, with low viral loading resulting in the virus being difficult to spread. Recombinant adenovirus reduced disease score and viral shedding compared with FI-HSV-2 and acyclovir, and the addition of ΔUL25 further reduced the disease score and shedding compared to gD2 alone (Table 2 and Table 3).

### 3.5. Antibody Response in HSV-2 Infected Guinea Pigs

**Figure 4:**
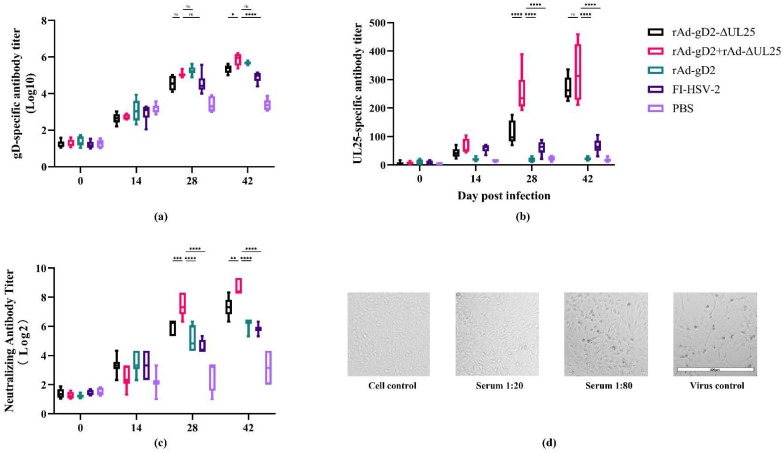
Antibody response to HSV-2-infected guinea pigs immunized with vaccines from different time points. (**a**) gD-specific antibody titer of guinea pigs was determined by direct ELISA. *Y*-axis shows IgG antibody titers in the log10 scale. rAd-gD2 + rAd-ΔUL25 was the highest in gD-specific antibody titer compared with rAd-gD2ΔUL25 and rAd-gD2. Because of the challenge, the PBS control group also developed a detectable gD-specific antibody, while all the other groups were significantly higher than PBS. (**b**) UL25-specific antibody titer of guinea pigs was determined by direct ELISA. *Y*-axis shows UL25-specific antibody titers. rAd-gD2 + rAd-ΔUL25 was the highest in gD-specific antibody titer and was non-significant (ns) with rAd-gD2ΔUL25. (**c**) Neutralizing antibody titer of guinea pigs was defined as the reciprocal value of the highest serum dilution for which no CPEs were detected. *Y*-axis shows neutralizing antibody titers in the log2 scale. rAd-gD2 + rAd-ΔUL25 was the highest in neutralizing antibody titer, approximately 3.5 times higher than rAd-gD2ΔUL25. All the immunized groups were significantly higher than PBS. **** *p* < 0.0001; *** *p* < 0.001; ** *p* < 0.01; * *p* < 0.05; ns, not statistically significant. (**d**) Images of CPE with different serum dilutions in rAd-gD2 + rAd-ΔUL25 (42 dpi).

gD- and UL25- specific IgG levels were determined using a direct ELISA. As shown in Figure 4a, both the recombinant adenovirus groups induced a higher titer of gD-specific antibody compared to the mock-immunized control group. Owing to the HSV-2 challenge, a gD-specific antibody titer was also induced in the PBS group. Among them, animals administered rAd-gD2 + rAd-ΔUL25 induced the highest gD-specific antibody titer, significantly higher (39%) than that of animals administered PBS; the titers of groups administered rAd-gD2ΔUL25, rAd-gD, and FI-HSV were 34%, 38%, and 29% higher, respectively, than that of the group administered PBS. The gD-specific antibody in rAd-gD2 + rAd-ΔUL25 was a little higher than in rAd-gD2ΔUL25 at 42 dpi, which may be the epitope masking of a fusion protein. Above all, all of our immunization groups could induce a high titer of gD-specific antibodies.

UL25-specific antibody titers are shown in Figure 4b. The titer of the UL25-specific antibody was a little higher in rAd-gD2 + rAd-ΔUL25 than rAd-gD2ΔUL25 at 28 dpi but non-significant at 42 dpi. All the fusion forms and combination forms induced UL25-specific antibody titer after the two immunizations. Group administered FI-HSV-2 also induced few UL25-specific antibodies. These results indicated that our recombinant adenovirus vaccines could induce a high titer of gD-specific antibodies and a weak titer of UL25-specific antibodies.

A microneutralization assay was used to measure the anti-HSV-2 neutralizing antibody titer. As shown in Figure 4c,d, rAd-gD2 + rAd-ΔUL25 significantly induced the highest titer of neutralizing antibody (*p* < 0.0001), 3.5 times higher than rAd-gD2ΔUL25, 5.2 times higher than rAd-gD2, 6.8 times higher than that of FI-HSV-2, respectively. Relative to the challenge, the PBS-immunized group also developed a detectable antibody titer. rAd-gD2ΔUL25 induced a significantly higher titer of neutralizing antibody than rAd-gD2, indicating that adding UL25 as the immunogen could significantly increase the titer of neutralizing antibody in HSV-2 infected guinea pigs, and the combination form could induce higher neutralizing antibody titers than fusion form.

### 3.6. Cellular Response in HSV-2 Infected Guinea Pigs

**Figure 5:**
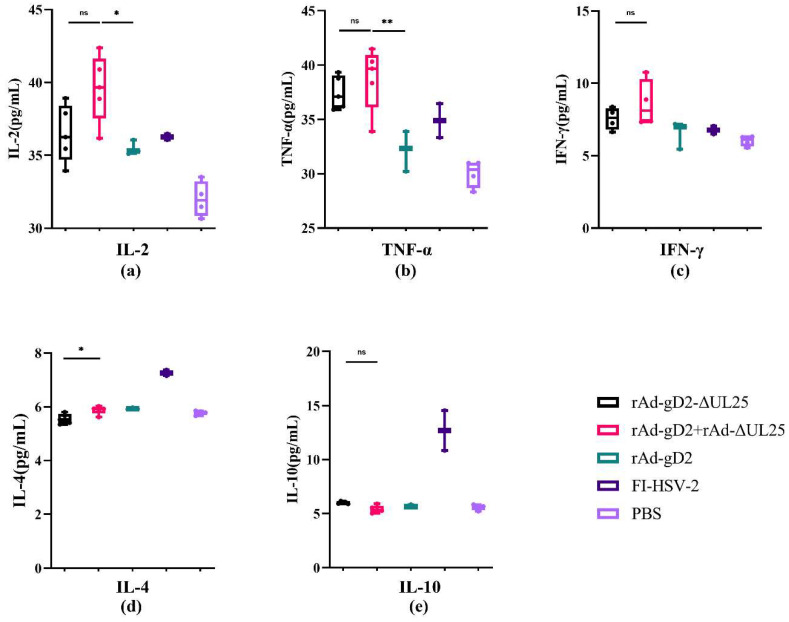
Cellular response to HSV-2-infected guinea pigs immunized with vaccines. Guinea pigs were evaluated for their Th1 (IL-2, TNF-α, and IFN-γ) and Th2 (IL-4 and IL-10) cytokines levels in serum. The secretion of cytokines IL-2 (**a**), TNF-α (**b**), IFN-γ (**c**), IL-4 (**d**), and IL-10 (**e**) in serum was measured by ELISA. * *p* < 0.05; ** *p* < 0.01; ns, not statistically significant.

To evaluate the cellular response induced by vaccines, we examined Th1 (IL-2, TNF-α, and IFN-γ) and Th2 (IL-4 and IL-10) cytokine levels in guinea pig serum using ELISA. The results are shown in Figure 5. Because of the higher dose of immunization, the rAd-gD2 + rAd-ΔUL25 immunized group induced slightly higher levels of the Th1-type cellular immune response than the rAd-gD2ΔUL25 immunized group, although the difference was not significant. The rAd-gD2 + rAd-ΔUL25 immunized group induced significantly higher levels of the Th1-type cellular immune responses than rAd-gD2, indicating the enhancement of cellular immunity by rAd-ΔUL25. When considering the Th2-type cellular responses, rAd-gD2 + rAd-ΔUL25 induced significantly higher levels of IL-4 secretion than rAd-gD2ΔUL25 did (*p* = 0.0174). The FI-HSV-2 group showed the highest level of Th2 immune response, as observed in our previous results [14,20]. These results indicate that our recombinant adenovirus vaccines induced a Th1-type cellular immune response in guinea pigs.

## 4. Discussion

The socioeconomic and economic burden, in addition to the high incidence rate caused by recurrent GH, emphasizes the need for developing therapeutic herpes vaccines [21]. A therapeutic vaccine should be designed to prevent symptomatic recurrences, control asymptomatic viral shedding, and prevent disease transmission [18,22]. Therefore, there is an urgent need to develop effective therapeutic vaccines. HSV-2-based glycoprotein-subunit vaccines failed to provide adequate antiviral protection in large-scale clinical trials [23] as a result of the virus developing strategies to evade immune responses.

Over the past 20 years, there have been several clinical trials performed that used HSV-2 glycoproteins essential for virus entry as immunogens, but none achieved its primary endpoint [9]. Glycoprotein D is a common candidate target for HSV-2 vaccines and is widely used in clinical trials [24,25,26]. However, gD only elicits neutralizing antibodies, weakly induces a cellular immune response, and has no functionalities (complement-dependent cytolysis, ADCC, ADCP) [27]. Experiments with the candidate therapeutic HSV-2 vaccine GEN-003 encouraged us to use T-cell-response proteins, such as capsid proteins, as candidate vaccines [14,15,16]. UL25, which is the dominant target of CD8 + T cell response in HSV-2-infected individuals, is an attractive target in HSV-2 therapeutic vaccines [17]. Adenovirus-based vaccines have been widely used in HSV research, focusing primarily on prophylactic vaccines in mouse models [28,29,30,31]. The advantage of adenovirus-based vaccines is their ability to produce multipurpose CD4 + and CD8 + T cells, as well as memory effector CD8 + T cells, which circulate in the body for over two months [32]. Adenovirus-based vaccines were found to be potent in preventing HSV-2 infection in animal models [30,33,34].

In our previous studies, the recombinant adenovirus vaccine, rAd-gD2ΔUL25, was shown to confer a higher survival rate in an HSV-2 lethal dose-challenged mouse model than the rAd-gD2 and the rAd-ΔUL25 immunized groups did, via eliciting Th1-type immunity and elevating IFN-γ and IL-2 levels [1]. rAd-ΔUL25 induced UL25-specific IFN-γ, and rAd-gD2 induced gD2-specific IFN-γ. However, the two immunized groups both showed weak protection, indicating more than one immune function may correlate with protection [9,29].

Several studies have shown that IFN-γ is important for the resolution of lesions and clearance of HSV-2 genital infections [35,36,37], with IL-2 being secreted within hours after CD8 + T cell activation [38]. Considering the ability of recombinant adenovirus vaccines to induce cellular immunity and the importance of T cell immune responses in controlling viral reactivation and HSV disease, this study evaluated a recombinant adenovirus as an HSV-2 therapeutic vaccine. Because of the lack of recurrence and shedding of reactivated viruses, mouse models cannot be used in therapeutic vaccines. In contrast, the guinea pig model represents the gold standard model to evaluate therapeutic vaccines since HSV-2-infected guinea pigs can develop pathological features of recurrence that mimic human disease [39]. However, a limitation of guinea pigs in evaluating recurrent HSV disease is the diminishing number of recurrences and recurrent shedding over time. The optimal observation period was 7–8 weeks after infection. Therefore, the guinea pig model cannot accurately predict the outcomes of clinical trials [40].

In this study, recombinant adenovirus-based vaccines were evaluated as therapeutic vaccines using a guinea pig model. Guinea pigs were challenged with an LD_50_ of HSV-2 and immunized with gD2 and ΔUL25 using two different approaches: fusion expressed (rAd-gD2ΔUL25) and combination expressed (rAd-gD2 + rAd-ΔUL25) vaccines. These adenovirus vaccines significantly improved the survival rate of guinea pigs compared to that of guinea pigs that received rAd-gD2 alone (Figure 2), which was similar to the findings of previous studies in mouse models. These results indicated that the UL25 addition improved the survival of both prophylactic and therapeutic HSV-2 vaccines.

In humoral immunity, after the challenge, all the groups induced specific IgG, and neutralizing antibodies were induced in all groups. The recombinant adenovirus vaccines evaluated in this study induced higher titers of both specific IgG and neutralizing antibodies than FI-HSV-2 did. The combination expressing group (rAd-gD2 + rAd-ΔUL25) achieved better neutralizing antibody titers than the fusion expressing group (rAd-gD2ΔUL25) (Figure 4), indicating that combined immunization with two different antigens induced a higher level of humoral immunity than fused immunization did [15]. Similar results were found in VZV, EV71, and CAV16 vaccines [41,42], which may be due to the masking of neutralizing epitopes on fusion-expressed proteins. In our future studies, we will analyze the differences in epitope in fusion and combination adenoviruses.

Although the combination-expressed group induced significantly higher levels of immunity than the fusion-expressed group did, both vaccines had protective potential, indicating that cellular response is crucial to controlling disease recurrence. ADCC and antibody-mediated phagocytosis also play an important role in immune protection [43,44,45,46]. Since the infection occurs on mucosal surfaces, sIgA may also be crucial to immune protection, which will be verified in our future studies.

Compared with the FI-HSV-2 or rAd-gD2 groups, the addition of UL25 in the fusion expressed and in combination expressed groups reduced the symptoms at recurrence and the amount of virus in the genitals, which affected transmission [18]. Our previous study showed that the addition of UL25 significantly elevated the cellular response in mouse models to both adenovirus and DNA vaccines [14,20]. T-cell response plays an important protective role against HSV-2 infection [25]. Here, all adenovirus vaccination groups induced Th1 type T-cell response after two immunizations, similar to recent studies [47,48]. The combination-expressed group (rAd-gD2 + rAd-ΔUL25) induced a significantly higher level of the Th1-type immune response, indicating the enhancing effect of rAd-ΔUL25 in inducing a cellular immune response. These results were also observed in our previous study using a mouse model [14]. In both mice and guinea pigs, the addition of ΔUL25 significantly induced a Th1-type immune response. In combination with the results of survival rate, the Th1-type immune response plays an important role in HSV-2 recurrence in a guinea pig model, and the protective effect caused by the addition of UL25 may be due to Th1-type immunity or other functionalities (complement-dependent cytolysis, ADCC, and ADCP), which will be verified in our further studies.

Many studies showed that combining different repertoires of viral antigens may help mount a protective immune response [19,49,50]. The results of our study are consistent with those of Christopher et al. [15], who reported that the addition of UL5 and UL30 to gD2 DNA improved the survival rate in guinea pigs. Zhang et al. [51] reported that *env-gag* mRNA vaccines induced higher immunogenicity than *env* vaccines alone, suggesting that the addition of selected essential proteins for viral replication together with glycoproteins could induce a stronger immune response.

In HSV-2 vaccines, UL25 is one of the most frequently recognized HSV-2-specific CD8 + T cell epitopes in HSV-2 seropositive individuals and is a potent immunogen for conferring protective immunity [52]. Srivastava et al. [39] showed that challenged guinea pigs were not protected with UL25 protein. These results indicated that the synergistic effect of UL25 and gD2 improved the survival rate of the guinea pigs. The addition of UL25 prevented viral transmission and reduced symptoms at the time of disease recurrence. These results further illustrated the importance of cellular immunity in the prevention of disease recurrence and transmission.

The addition of UL25 to gD2 in both the fusion expressed and combination expressed vaccines could provide protection in the guinea pig model. In this study, the recombinant vaccines were shown to be superior to FI-HSV-2 in inducing gD-specific antibodies and neutralizing antibodies. Moreover, the recombinant vaccines were superior to acyclovir in preventing recurrence, hence posing a potential therapeutic vaccine against recurrent infections.

## Figures and Tables

**Table 1:** Immunized with recombinant adenovirus-based vaccine.

Immunogen	Immunization Mode	Immunization Dose
rAd-gD2ΔUL25	i.m.	1 × 10^8^ PFU
rAd-gD2 + rAd-ΔUL25	i.m.	1 × 10^8^ PFU + 1 × 10^8^ PFU
rAd-gD2	i.m.	1 × 10^8^ PFU
FI-HSV-2	i.m.	12.5 μg
Acyclovir	p.o.	15 mg/kg
PBS	i.m.	100 μL

**Table 2:** Effect of adenovirus vaccines on recurrent lesion score in HSV-2-infected guinea pigs.

Groups	14–27 dpi		28–41 dpi		42–56 dpi	
	Recurrent Mean Lesions		Recurrent Mean Lesions		Recurrent Mean Lesions	
	Score	% Reduction ^a^	Score	% Reduction ^a^	Score	% Reduction ^a^
PBS	12.0 ± 3.1		11.0 ± 1.2		11.0 ± 2.2	
Acyclovir	10.6 ± 1.4	11.6%	9.3 ± 2.9	15.5%	7.3 ± 1.2	33.6%
FI-HSV-2	11.3 ± 1.5	5.8%	10.2 ± 1.6	0.7%	7.7 ± 4.2	30.0%
rAd-gD2	10.2 ± 1.7	15.0% ^b^	6.8 ± 1.5	38.2% ^b^	3.0 ± 2.4	72.7% ^c^
rAd-gD2 + rAd-ΔUL25	4.8 ± 3.8	60.0% ^c^	1.5 ± 1.1	86.4% ^d^	1.3 ± 0.5	88.2% ^c^
rAd-gD2ΔUL25	3.5 ± 3.2	70.8% ^d^	1.5 ± 1.2	86.4% ^d^	1.6 ± 0.9	85.5% ^c^

Dpi, day post-infection. ^a^ % Reduction, percent reduction compared to the PBS control. ^b^ *p* < 0.05 compared to the PBS control group. ^c^ *p* < 0.01 compared to the PBS control group. ^d^ *p* < 0.001 compared to the PBS control group.

**Table 3:** Effect of adenovirus vaccines on viral shedding in HSV-2-infected guinea pigs.

Recurrent Viral Shedding								
Groups	14–27 dpi		28–41 dpi		42–56 dpi		Total	
	Days ^a^	Quantity ^b^	Days	Quantity	Days	Quantity	Days	Quantity
PBS	14	2.5 ± 1.2	14	2.5 ± 0.7	12	3.5 ± 1.5	40	2.8 ± 1.1
Acyclovir	10	1.7 ± 1.0	5	1.3 ± 0.4	10	2.0 ± 0.9	25	1.7 ± 1.3
FI-HSV-2	10	0.6 ± 0.4	9	1.6 ± 0.6	13	1.5 ± 0.5	32	1.2 ± 0.6
rAd-gD2	9	0.9 ± 0.2	4	1.8 ± 1.3	13	1.7 ± 1.0	26	1.4 ± 1.1
rAd-gD2 + rAd-ΔUL25	4	1.0 ± 0.6 ^e^	1	1.0 ± 0.5 ^e^	6	1.0 ± 0.5 ^d^	11	1.0 ± 0.6 ^f^
rAd-gD2ΔUL25	6	1.2 ± 0.5 ^d^	1	1.5 ± 0.5	5	1.5 ± 1.3 ^c^	12	1.4 ± 0.7 ^f^

^a^ Days, any animal in the groups showed a positive copy of HSV-2 DNA is considered a recurrence viral shedding day. ^b^ Quantity, mean Log10 DNA copies. ^c^ *p* < 0.05 compared to the PBS control group. ^d^ *p* < 0.01 compared to the PBS control group. ^e^ *p* < 0.001 compared to the PBS control group. ^f^ *p* < 0.0001 compared to the PBS control group.

## Data Availability

All data, models, and code generated or used during the study appear in the submitted article. The data that support the findings of this study are openly available in Mendeley Data, V1, http://doi.org/10.17632/hd962mb4rh.1.
